# Internal Mammary Recipient Site Breast Cancer Recurrence Following Delayed Microvascular Breast Reconstruction

**Published:** 2013-01-21

**Authors:** Anais Rosich-Medina, Susan Wang, Ertan Erel, Charles M. Malata

**Affiliations:** ^a^Department of Plastic and Reconstructive Surgery, Addenbrooke's Hospital, Cambridge; ^b^Clinical School of Medicine, Cambridge University; ^c^Cambridge Breast Unit, Addenbrooke's Hospital, Cambridge University Hospitals NHS Foundation Trust, Hills Road, Cambridge, United Kingdom

## Abstract

**Objective:** The internal mammary vessels are a popular recipient site for microsurgical anastomoses of free flap breast reconstructions. We, however, observed 3 patients undergoing internal mammary vessel delayed free flap breast reconstruction that subsequently developed tumor recurrence at this site. We reviewed their characteristics to determine whether there was a correlation between delayed microsurgical reconstruction and local recurrence. **Methods:** A retrospective review of a single surgeon's delayed free flap breast reconstructions using the internal mammary vessels was conducted over a 7-year period to identify the time intervals between mastectomy and delayed breast reconstruction and between delayed breast reconstruction and recurrence. **Results:** Three patients developed local recurrence at the site of the microvascular anastomoses following delayed breast reconstruction. All patients had been disease-free following mastectomy. The median time interval between mastectomy and delayed breast reconstruction was 28 months (range = 20-120 months) while that between delayed breast reconstruction and local recurrence was 7 months (range = 4-10 months). Two patients died from metastatic disease, 36 and 72 months following their local recurrence. One patient remains alive 44 months after reconstruction. **Conclusions:** Local tumor recurrence at the internal mammary vessel dissection site following delayed breast reconstruction raises the question whether these 2 events may be related. Specifically, could internal mammary vessel dissection undertaken for delayed microsurgical reconstruction predispose to recurrence in the internal mammary lymph nodes? Further research is needed to ascertain whether delayed breast reconstruction increases the risk of local recurrence in this patient group.

Autologous free flap breast reconstruction is considered by many to be the optimum technique for breast reconstruction. Worldwide the use of the internal mammary vessels (IMVs) as recipients for microsurgical anastomoses has gained popularity in recent years.^1^^-^3

There have been recent concerns regarding an increased risk of local, regional, and systemic recurrence following immediate breast reconstruction.^4^^-^6 The possible mechanisms for this remain to be elucidated. In contrast, the literature on the association between delayed breast reconstruction (DBR) and local cancer recurrence is sparse consisting mostly of letters and small case series.^7^^,^^8^ However, a recent retrospective cohort study of 125 patients conducted in Sweden showed a statistically significant higher risk of locoregional and distant recurrent disease following DBR with microvascular flaps and pedicled musculocutaneous flaps than those with mastectomy alone.^9^

The present case series presents 3 previously disease-free patients who developed local recurrence on the chest wall at the site of the microvascular anastomoses following DBR.

## METHODS

A retrospective review of the senior author's (C.M.M.) delayed free flap breast reconstructions using the IMV as the recipient site was conducted between January 2004 and January 2011. All patients who developed local breast cancer recurrence at the site of the IMV anastomoses following DBR were identified from the paper and electronic medical records. Their demographics, reconstructive details, histology reports, reconstructive and oncology outcomes, and follow-up were reviewed. The 3 main areas of interest were (1) the time interval between mastectomy and DBR, (2) the time interval between DBR and local internal mammary recurrence, and (3) patient survival and follow-up.

## TECHNIQUE OF IMV ANASTOMOSES

Prior to 2008, the IMV dissection was performed using the traditional method of excising the third costal cartilage and all the intercostal muscles between the second and fourth costal cartilages. From May 2008, all patients underwent vessel exposure using the total rib-preservation technique.^3^ Any incidental lymph nodes encountered during vessel exposure are also opportunistically harvested.^10^ End-to-end vessel anastomoses were all performed using 9/0 monofilament nylon. The venous coupler was routinely employed starting January 2011.

## RESULTS

A total of 68 delayed lower abdominal free flaps were performed by the senior author during the study period. Three patients developed local chest wall recurrence at the site of IMV anastomosis following DBR, thus giving an internal mammary lymph node (IMLN) recurrence rate of 4.4% (3/68). The histologies of the local recurrences in all 3 patients were the same as the original breast cancer diagnosis.

The 3 index patients were nonsmokers, and, interestingly, all had right-sided invasive ductal carcinomas, which had been treated with mastectomy and axillary node clearance. Their mean age at the time of primary breast cancer diagnosis was 44.7 years (range = 39-49) (Table 1). All 3 women were staged T2N1M0 preoperatively and 2 patients received adjuvant chemotherapy and radiotherapy, while one patient received preoperative radiotherapy following a previous wide local excision. Two of the 3 patients were oestrogen receptor positive (ER+) and received hormonal therapy with tamoxifen. The 3 patients were cleared oncologically (mammography and clinical follow-up) prior to their breast reconstructions and the histology of the mastectomy scars was negative in all cases. Incidental lymph nodes were harvested during vessel exposure in 1 patient. There was no evidence of metastatic malignancy histologically.

**Figure 1:**
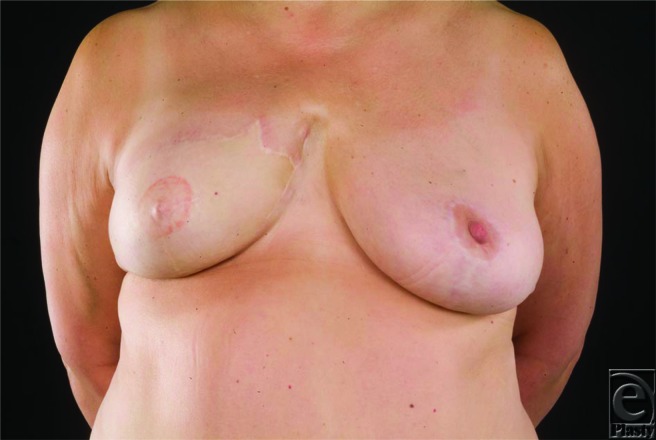

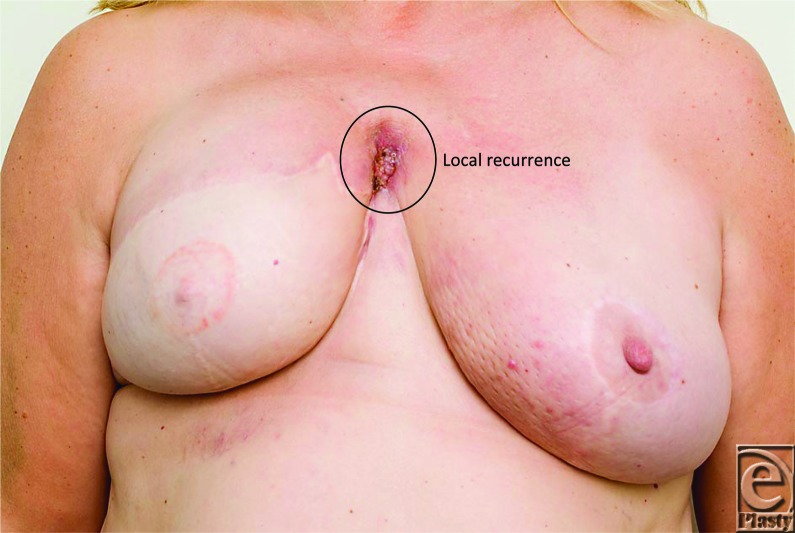

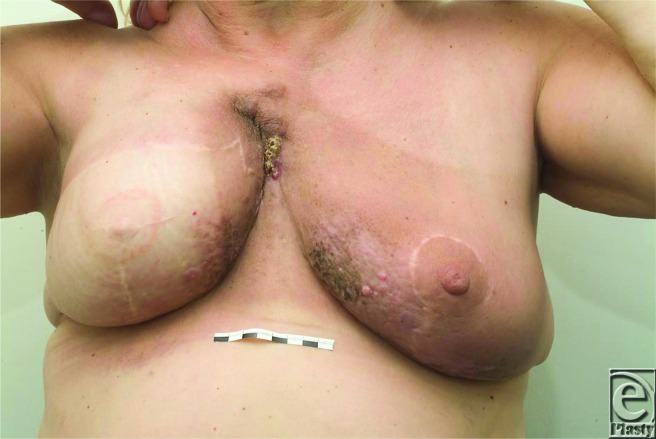
(*a*). Patient C following delayed right-sided breast reconstruction with a free TRAM flap and a contralateral balancing mastopexy. (*b*). After 10 months of reconstruction, patient C developed a fungating recurrence at the internal mammary anastomotic site and required treatment with radiotherapy. (*c*). Patient C following radiotherapy.

**Figure 2:**
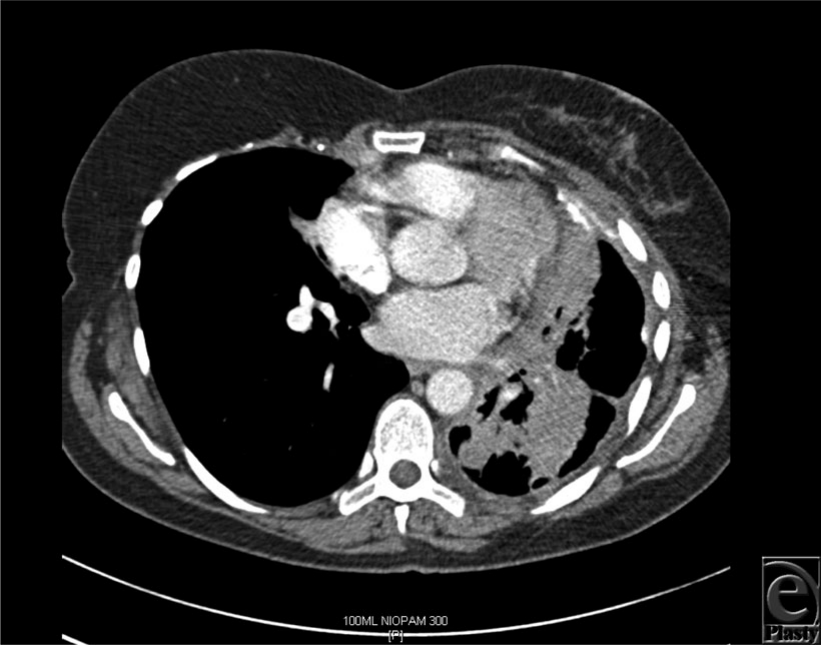
Computed tomographic scan of patient A demonstrating intrapleural metastases, intrapulmonary nodes, and thickening of the left pleural surface.

The median time interval between mastectomy and DBR was 28 months (range = 20-120 months), whereas the median time interval between DBR and local recurrence was 7 months (range = 4-10 months). Figures 1a to 1c show the reconstruction, recurrence, and postradiotherapy status of patient C. The patient with the longest time interval between mastectomy and DBR (120 months) developed chest wall recurrence only 4 months after her reconstruction (patient A). She also developed intrapleural metastases and intrapulmonary nodes (Fig 2). She subsequently developed bone and peritoneal metastases and is currently receiving palliative treatment but remains alive 44 months following her recurrence. The other 2 patients have died as a consequence of metastatic disease, 36 and 72 months following their local recurrence.

## DISCUSSION

The 3 patients in our case series developed local recurrence at the site of microvascular anastomosis within 10 months of their DBR, despite being disease-free for a long time following their mastectomy. In fact, one of the patients was disease-free for 10 years prior to her DBR and then presented shortly (4 months) after her reconstruction with local intercostal space recurrence. This temporal relationship between the DBRs and the recurrences therefore raises the question whether an aspect of the DBR such as the IMV dissection or general immunosuppression may have in some way stimulated the local breast cancer recurrence. Could it have activated dormant tumor cells present in the lymph nodes at the internal mammary dissection site present for instance in the IMLNs? It has previously been documented that surgical trauma and other stress factors can “activate dormant micrometastases” and this may be a potential explanation for the local recurrence in our patients.^11^ It is also conceivable that breast cancer cells which had metastasized to the IMLNs and lymphatics might have been inactive prior to the reconstructive surgery but were reactivated following the reconstruction. The patient who had incidental lymph nodes harvested during vessel exposure was found not to have metastatic malignancy at the time of DBR, thus suggesting that there was no evidence of locoregional metastases at the time of DBR. This finding would suggest that surgical trauma or other stress factors during the DBR may have “re-activated dormant micrometastases,” which months later caused the patient to present with local chest wall recurrence.

There have been anecdotal reports of possible links between DBR and cancer recurrence.^7^^,^^8^ Most reports have focused on the possible links between immediate breast reconstruction and local recurrence.^4^^-^5,12^-^15 However, a recent study comparing recurrence rates in women with delayed large flap (postmastectomy) breast reconstruction with mastectomy alone found a significantly higher risk of breast cancer recurrence following DBR.^9^ Our study lends further credence to this association. Despite this, the mechanism by which DBR can influence local breast cancer recurrence remains to be elucidated.

Several theories have been proposed for the role of surgery in precipitating local breast cancer recurrence. First, the insult of major surgery may upset the delicate balance between the immune system and dormant breast cancer cells.^9^ Patients with breast cancer are known to harbor micrometastases at different sites and surgical trauma may reactivate the dormant micrometastases, which could result in early local recurrence or distant metastases.^11^ Second, mechanical dispersal of dormant tumor cells in the IMLNs during IMV exposure is a possibility. Disruption of metastatic growth suppression may lead to uncontrolled cancer growth in tissues where the metastatic cells had been previously suppressed and not clinically apparent. In contrast to flap reconstruction, DBR with implants did not increase breast cancer recurrence rates after reconstruction.^16^ This perhaps suggests that it may not be the surgical trauma per se that may be implicated but other factors such as disruption of lymphatics.

The case series herein reported raises the question whether there may be a causal link between DBR and local recurrence following microsurgical breast reconstruction or whether our findings were coincidental. If there was a causal link, this would have important implications for the use of the internal mammary recipient site for microsurgery. For the reconstructive microvascular surgeon, this would create a dilemma whether to preferentially use this site which has multiple advantages over the subscapular-thoracodorsal vascular axis or avoid it for fear of “reactivating” recurrence.^17^

## CONCLUSION

Although no generalizations can be made from our small case series, we would like to make other clinicians aware that there is a theoretical possibility of a correlation between delayed free flap breast reconstruction using the IMV as the recipient site and the onset of local breast cancer recurrence. We suggest that other clinicians with similar experience should formally report their results in the literature to generate more informative discussion.

## Figures and Tables

**Table 1:** Patient demographics, locoregional recurrence, and outcomes

Patient	Age	Histology, Grade, Receptor Status, TNM Staging	Time Between Mastectomy & DBR (mo)	Time Between DBR & Recurrence (mo)	Locoregional Recurrence	Outcome (mo)
A	39	Invasive ductal, grade II, ER+, HER2+, T2N1M0	120 mo	4 mo	Chest wall Intrapleural & intrapulmonary nodules	Bone & peritoneal metastases – alive 44 mo after local recurrence
B	46	Invasive ductal, grade II, PR+, T2N1M0	28 mo	7 mo	Chest wall, Right SCF	Died 72 mo after local recurrence
C	49	Invasive ductal, grade III, ER+, T2N1M0	20 mo	10 mo	Chest wall, Right SCF, Left axilla	Died 36 mo after local recurrence

DBR indicates delayed breast reconstruction; ER+, oestrogen receptor positive; HER2, human epidermal growth factor receptor; SCF, supraclavicular fossa; mo, months.
